# Recurrent Febrile Urinary Tract Infections in a Five-Year-Old Girl

**DOI:** 10.7759/cureus.14412

**Published:** 2021-04-11

**Authors:** Hesham H AbdelAziz, Mohamed H Gad

**Affiliations:** 1 Department of Urology, Al Soliman Hospital, Port Said, EGY; 2 Department of Urology, Medical University of Łódź, Łódź, POL

**Keywords:** urinary tract infection, vesicoureteral reflux, overactive bladder, detrusor overactivity, detrusor external sphincter dyssynergia, bladder outlet obstruction, spina bifida occulta

## Abstract

Urinary tract infections (UTIs) are one of the most common bacterial infections of childhood and in pediatric urology. Medical history, examination findings, and clinical course usually vary with the patient's age. Hence, there are no specific clinical features that are strictly associated with UTI in infants or children. This report presents a five-year-old female patient with spina bifida occulta and recurrent history of febrile UTIs diagnosed with detrusor sphincter dyssynergia over the last year. Urodynamic study confirmed an overactive uroflow pattern with bladder outlet obstruction (BOO) and high flow obstruction with long voiding time and terminal dribbling. The aim of this report is to showcase a typical presentation of secondary detrusor overactivity to bladder outlet obstruction in a patient with a coincidental finding of spina bifida occulta and to emphasize the importance of early treatment intervention in decreasing the risk of future complications such as UTIs.

## Introduction

Vesicoureteral reflux (VUR) and lower urinary tract dysfunction are directly correlated as children and especially females, with lower urinary tract dysfunction, tend to have a higher incidence of urinary tract infection (UTI) than males. It is well known that voiding dysfunction may have an etiologic factor to an extent in developing or even exacerbating VUR [1] and when it is associated with VUR can lead to increased risk of UTI. It is believed that the treatment of bladder dysfunction increases the likelihood of spontaneous resolution of reflux. Chronic increased bladder pressure from post-residual urine after incomplete voiding may lead to UTIs due to urinary stasis; therefore, it can lead to risk of deterioration of the upper urinary tract [2]. Thus, converting high-pressure bladder with outlet obstruction based on detrusor/sphincter dyssynergia into a low-pressure storage creates a safer milieu for the upper urinary tracts, and reducing the risk is the primary objective of management.

Spina bifida is a disorder in which posterior vertebral elements fail to fuse properly during development leading to a bifid osseous compartment, usually in lower lumbosacral spine. In this girl’s case, spina bifida occulta (SBO) was presented. It is a mild form of spina bifida, which is a defect in the vertebral arch of either L5 or S1 and results in failure of posterior vertebral arches to fuse. Although the defect occurs during development, some reported cases of SBO seemed to diminish with aging and bone maturity. It is important to remember that the vertebral body will articulate with the vertebral arch at the neurocentral joints at birth, and the two halves of the posterior arches fuse between the ages of five and eight years [3]. SBO was a coincidental finding, and it is already established in this report that no accompanying neurological abnormalities were noted.

Detrusor sphincter dyssynergia (DSD) is the urodynamic description of bladder outlet obstruction (BOO) from detrusor muscle contraction with concomitant involuntary external urethral sphincter activation. Presentation of clinical signs and symptoms of patients with neurogenic bladder dysfunctions associated with neurological origins such as SBO might not be recognized until the recurrence of UTIs, onset of urinary incontinence, and urinary retention and/or occurrence of orthopedic abnormalities during childhood. It is commonly diagnosed during voiding in urodynamic studies using voiding cystourethrograms.

Patients with detrusor overactivity in whom oral pharmacologic treatment has failed to normalize intravesical pressure are candidates for transurethral botulinum toxin injection to temporarily alleviate BOO. Urinary sphincterotomy has shown to be proven effective in initially decreasing voiding resistance and improving voiding efficiency, but the associated morbidity of this procedure is of a considerable amount [4].

As the SBO exhibited no orthopedic manifestations in this report and how generally SBO and VUR may be related to voiding dysfunctions, its significance in this case is not well established.

## Case presentation

A five-year-old girl presented to my care with recurrent episodes of recurrent febrile episodes of UTIs. Review of her medical history revealed multiple recurrent episodes of UTIs within the last year. She was referred to my care due to increased frequency and daytime urinary incontinence as well as foul-smelling urine since the age of four. Although she had a history of recurrent UTIs, she did not visit a urologist. Past instances were diagnosed clinically, based on the presence of dysuria and abdominal pain. At her initial visit to my office, she was normotensive, she did not look toxic, and physical examination of her abdomen and external genitalia was unremarkable. Her temperature was 39.0°C (102.2°F), and her developmental milestones were normal. Her past medical history revealed multiple episodes of constipation, and urinalysis revealed bacteriuria. Her blood tests showed a peripheral white blood cell count of 12.1 × 10^9^/L, normal hemoglobin, normal platelet count, and a high C-reactive protein of 60 μg/mL. She remained febrile for two days.

Initial uroflowmetry could not be performed due to low bladder volume. Filling cystometrogram demonstrated detrusor overactivity (DO) with a maximum flow of 12.3 ml/s at a urethral resistance (URA) of 22.25 cm H_2_O with detrusor pressures of 50.7 cm H_2_O. This was associated with long voiding time secondary to DO with a post-voiding residual of 39 ml. A pressure flow study showed a low flow during voiding phase with a maximum flow of 13.1 ml/s with an average voiding volume of 239.8 cc, post-voiding residual of 42 cc, and a maximum detrusor pressure of 17.2 cm H_2_O noted.

**Figure 1:**
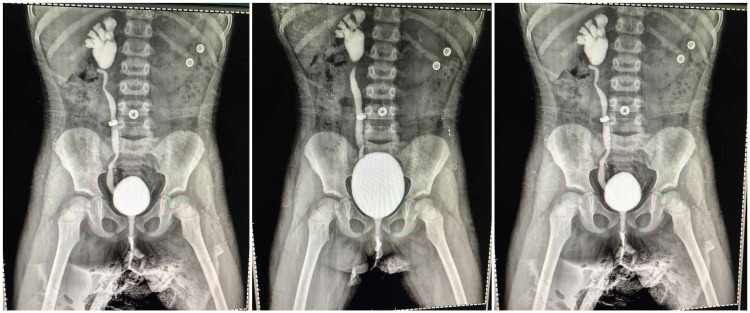
The collecting system of the right kidney with VUR revealing the "flowerpot" sign VUR, Vesicoureteral reflux.

A **voiding cystourethrogram (VCUG)** revealed dilated calyces cephalad looking like a dilated funnel-shaped renal pelvis, as well as high grade of right vesicoureteral reflux (VUR) and left-sided mild VUR. VCUG revealed the collecting system of the right kidney with VUR revealing the “flowerpot” sign in addition to a trabeculated bladder during detrusor overactivity with open bladder neck and patent urethra (Figure 1).

**Ultrasonography **revealed chronic pyelonephritic changes in the right kidney with dilated pelvicalyceal system and a normal left renal unit with a thickened bladder wall and significant post-voiding residual urine. 

**Technetium-99m dimercaptosuccinic acid (99mTc-DMSA)** renal scan showed evidence of cortical scar in single photon emission tomography (SPECT) images. The right kidney appeared small in size with poor intra-renal cortical fixation, high background activity, and homogeneous radiotracer uptake, keeping deteriorated functioning tubular renal mass therein. The left kidney appeared normal in size with a fair level of cortical fixation indicating a reasonable functioning tubular renal mass, and no evidence of cortical scarring was detected.

**Filling cystometrogram** demonstrated high amplitude detrusor overactivity (DO) starting within normal urinary bladder (UB) capacity for age with maximum cystometric bladder capacity (MCBC) of 280 ml. It also highlighted intact sensations and compliant UB near capacity with multiple, phasic, unprovoked detrusor overactivity and without leak till full capacity. Higher filling pressure and no urodynamic stress urinary incontinence (SUI) were observed at different UB volumes.

**Voiding cystometrogram** showed increased sphincter activity with a post-void residual (PVR) of 39 ml, and when the testing was redone, it still showed the same data of DO with long voiding time with a PVR of 39 ml. This was associated with obstructed voiding pattern with compensated detrusor contractility, resembling a detrusor-external sphincter dyssynergia (DESD)-like pattern. 

The patient was commenced on alpha-blocker (tamsulosin hydrochloride 0.4 mg, once daily), anticholinergic (solifenacin succinate 5 mg tid or bid), and long-term suppressive antimicrobial therapy (sulfamethoxazole and trimethoprim). Her follow-up included (1) urine specimen for culture and sensitivity, (2) ultrasonography for post-void residue, (3) cystometry for detrusor overactivity, and (4) nephrectomy to be considered once a low intravesicular bladder pressure is achieved; no residual urine presence and absence of any degree of left VUR during these three to six months of monitoring and follow-up.

## Discussion

UTIs are one of the most common bacterial infections of childhood and in pediatric urology. Medical history, examination findings, and clinical course usually vary with the patient's age. And so, there is no specific signs or symptoms that are strictly associated with UTI in infants or children. It is generally accepted, however, that diagnosing recurrent UTIs depends on the characteristic of the clinical course, past medical history, and nonspecific symptoms such as urinary frequency, urgency, abdominal and/or suprapubic discomfort, and dysuria [5]. Recurrence of febrile UTI after a satisfactory management and follow-up does require further laboratory diagnostics.

Performing a voiding cystourethrography (VCUG) after episodic recurrence of febrile UTI to undergo an evaluation for voiding dysfunction is indicated if renal and/or bladder ultrasonography reveals obstructive uropathy, scarring, and/or hydronephrosis or if children who were initially treatment responsive but afterward demonstrated an abnormal voiding pattern. However, there is a concern that with no performed VCUG after first reported febrile UTI, cases of VUR will eventually go undiagnosed [6].

Inadequate and lack of appropriate urological management are absolutely detrimental to upper urinary tract deterioration and renal damage caused by the high bladder pressures and hence the recurrence of UTIs. Approximately 48% of spina bifida patients with untreated urologic abnormalities have exhibited signs of kidney damage [7]. Deterioration associated with chronic BOO is directly correlated to inadequate compliance and high-storage pressures of the bladder; therefore, the finding of impaired contractility with BOO is an absolute indication for management. Therefore, voiding cystometrogram and voiding pressure flow study are required in evaluating the degree of potential obstruction [8]. The main objectives of urological management in spina bifida patients are (1) maintenance of a healthy upper urinary tract, (2) preservation of renal function, (3) resolving nocturnal enuresis to improve the patient’s overall quality of life, and (4) achieving continence.

Several studies have concluded that complications associated with upper tract deterioration are vesicoureteral reflux, increased intravesical pressures (low bladder compliance and/or detrusor muscle overactivity), DSD, and high leaking pressure capacity [9-11] that do warrant intervention with anticholinergic drugs to increase bladder capacity [12].

Yeung et al. have reported that an existing bladder dysfunction leads to a lower resolution rate of a VUR, and hence, abnormal bladder function is a predictable prognosis for persistence of reflux [13]. The intravesical pressures, which vary depending on the severity of functional disorder, can cause bladder disorders such as detrusor hyperreflexia, vesical compliance, persistent VUR, and post-voiding residual urine [14,15].

There are a multitude of factors contributing to the rate of recurrent UTI in children; these include recent continuous broad-spectrum antibiotic therapy/prophylaxis, anatomic abnormalities, VUR/voiding dysfunction, and bladder and bowel dysfunction (BBD). BBD in the setting of VUR results in a 56% probability of recurrent UTI compared to a 25.4% in pediatric patients with VUR [16]. And so, a comprehensive BBD treatment for patients affected by voiding dysfunction will likely result in 70% spontaneous VUR resolution compared to 40% and 38% for those with detrusor underutilization and idiopathic detrusor overactivity in mild-moderate VUR cases, respectively [17].

Antibiotic prophylaxis in reducing the risk of UTI recurrence in patients with bladder dysfunction and VUR was shown to be particularly effective (hazard ratio [HR] 0.21, 95% CI, 0.08-0.58) and in children whose index infection was febrile (hazard ratio 0.41, 95% CI, 0.26-0.64) [18].

The outlet obstruction based on detrusor/sphincter dyssynergia has been managed chronically with clean intermittent catheterization (CIC) and anticholinergics. Anticholinergics increase bladder capacity and provide low vesical pressures for bladder storage capacity. Anticholinergics such as oxybutynin are cost-effective and can be easily administered either orally or intravesically. Therefore, CIC is needed to empty the bladder to achieve low-pressure storage and bypass the outlet obstruction. Geraniotis et al. have proven that CIC reduces the risk of upper urinary tract deterioration compared to conservative management [19].

However, urologists managing spina bifida patients are aware that no sustainable therapeutic effect can be achieved from long-term anticholinergic therapy either from withdrawal of medication or nonadherence to the long-term treatment plan. Ab et al. reported that in non-compliant patients, detrusor overactivity eventually recurred shortly after discontinuation of anticholinergics in 73% of the patients [20].

Intra-sphincteric injection therapy of the bladder with 100 U of botulinum toxin, Botox®, can be an alternative to antimuscarinic therapy. This therapy effectively suppresses detrusor contractions for six to nine months and needs to be repeated for every six to nine months. No adverse effects were reported in this patient. The long-term effects of the Botox injection are yet to be established in this patient’s case.

## Conclusions

Renal function preservation and early resolution of nocturnal enuresis are imperative in improving the quality of life for spina bifida patients with recurring UTIs. This patient’s multiple UTIs are mainly due to intravesical pressure from post-residual urine after incomplete voiding. Consequently, converting high-pressure bladder with outlet obstruction based on detrusor/sphincter dyssynergia into a low-pressure storage that is safe for the upper urinary tracts and reduces the risk is the mainstay of treatment. As there is no lasting therapeutic effect of long-term anticholinergics in suppression of detrusor overactivity in detrusor/sphincter dyssynergia, it is safe to assume DESD is primary of neuropathic origin.
